# Effects of dimethyl itaconate on expressions of NGFI-A and NGFI-B and inflammatory cytokines in the spinal cord in the formalin test

**DOI:** 10.1093/braincomms/fcae397

**Published:** 2024-11-11

**Authors:** Kaveh Rahimi, Mohammad Abbaszadeh, Sharareh Bakhtazad, Zohreh Ghotbeddin

**Affiliations:** Department of Basic Sciences, Faculty of Veterinary Medicine, Shahid Chamran University of Ahvaz, Ahvaz, Iran; Department of Basic Sciences, Faculty of Veterinary Medicine, Shahid Chamran University of Ahvaz, Ahvaz, Iran; Department of Basic Sciences, Faculty of Veterinary Medicine, Shahid Chamran University of Ahvaz, Ahvaz, Iran; Department of Basic Sciences, Faculty of Veterinary Medicine, Shahid Chamran University of Ahvaz, Ahvaz, Iran; Stem Cells and Transgenic Technology Research Center, Shahid Chamran University of Ahvaz, Ahvaz, Iran

**Keywords:** dimethyl itaconate, NGFI-A, NGFI-B, cytokines, formalin test

## Abstract

Neural sensitization can cause neuroinflammation, which is a type of inflammation that occurs in both the peripheral nervous system and central nervous system. The purpose of this study was to investigate the effect of dimethyl itaconate (DMI) on the expression of NGFI-A and NGFI-B and inflammatory cytokines in the spinal cord in the formalin test. The rats were divided into five groups: control, formalin, DMI 10 mg/kg + formalin, DMI 20 mg/kg + formalin and diclofenac sodium 10 mg/kg + formalin. We evaluated the impact of DMI on the spinal cords NGFI-A and NGFI-B expressions and inflammatory and anti-inflammatory cytokines [interleukin-1 beta (IL-1β), tumour necrosis factor-alpha (TNF-α), interleukin-6 (IL-6) and interleukin-10 (IL-10)]. The findings indicate that DMI 10, DMI 20 and diclofenac sodium 10 mg/kg can relieve pain in rats during the formalin test. In addition, these substances were found to reduce the expression of NGFI-A and NGFI-B in the spinal cord. Moreover, DMI 10, DMI 20 and diclofenac sodium 10 mg/kg were observed to increase the expression of IL-10 while decreasing IL-1β, TNF-α and IL-6 in the spinal cord when compared with the formalin group. We have found that administering DMI can alleviate pain in rats during formalin test. Through our research, we have observed that DMI decreases the expression of NGFI-A and NGFI-B in the spinal cord. Furthermore, DMI has been shown to increase the levels of IL-10 while decreasing IL-1β, TNF-α and IL-6 in the spinal cord.

## Teaser

Rahimi *et al*. reported that dimethyl itaconate (DMI) has an antinociceptive effect on the formalin test in rats. DMI can relieve pain in rats during the formalin test. DMI reduced the expression of NGFI-A and NGFI-B in the spinal cord. DMI increased anti-inflammatory cytokine and decreased inflammatory cytokine in the spinal cord.

## Graphical Abstract

**Graphical Abstract:**
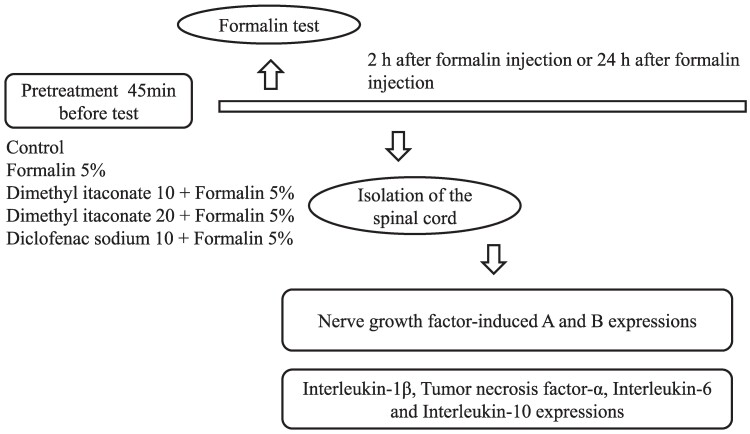

## Introduction

The innate immune response is a crucial defence mechanism against harmful agents. Cytokines such as tumour necrosis factor-alpha (TNF-α) and interleukin-1 beta (IL-1β) activate this response.^1^ When macrophages circulating in the body detect inflammatory substances, they secrete TNF-α, which triggers the innate immune response. TNF-α also stimulates macrophages to produce and release IL-1β in a paracrine manner.^2^ IL-1β, in turn, induces the release of interleukin-6 (IL-6) and causes the expression of intercellular adhesion molecule-1 in the affected area. These and other products induced by TNF-α and IL-1β work together to mediate the innate immune response.^3^ In contrast, interleukin-10 (IL-10) and transforming growth factor beta (TGF-β) are cytokines with anti-inflammatory effects that suppress the expression of other cytokines like IL-1β, IL-6 and TNF-α by suppressing macrophages.^4^ It has been demonstrated that peripheral inflammation leads to increased expression of inflammatory cytokines in the central nervous system. As a result, neuroinflammation may occur following peripheral inflammation.^5^ There is increasing evidence indicating that neuroinflammation, defined by the infiltration of immune cells, activation of glial cells and production of inflammatory mediators in both the peripheral and central nervous systems, plays a crucial role in both the development and persistence of chronic pain.^6^

Sensory neurons responsible for pain signals, known as nociceptors, rely on nerve growth factor (NGF) for their development and functional regulation. NGF is known to induce long-term thermal and mechanical hyperalgesia in animals and humans. Recently, humanized blocking antibodies targeting NGF have been tested in clinical trials for conditions like osteoarthritis, low back pain and interstitial cystitis and have shown significant analgesic potency. This suggests that anti-NGF drugs could revolutionize the treatment of chronic pain. It is, therefore, crucial to better understand the pathways and mechanisms controlled by NGF that are involved in initiating and sustaining thermal and mechanical hyperalgesia.^7^ NGF-induced genes (NGFI-A and NGFI-B) are a marker for neuronal activity after acute and chronic pain.^8-10^ The mRNA of these genes increased 60 min after formalin injection and then slowly decreased. The injection of formalin in the hind paw leads to increased early and late expression of many genes in the spinal cord.^11^ NGFI-A and NGFI-B are immediate early gene/transcription factors.^12^

Dimethyl itaconate (DMI) (2-methylidene butanedioic acid) is a bio-based unsaturated di-carbonic acid with a crystalline structure and white color.^13^ Low doses of DMI have no effect on neuronal viability, but high doses may be toxic to neurons.^14^ Previously, researchers have examined the analgesic effects of itaconate administered intraperitoneally and spinally in male and female rats with chronic nerve injury. They found that applying 4-octyl itaconate to the spine reduced the nerve responses in the injured rats. Through molecular biology experiments and studies in mice, they discovered that itaconate's pain-relieving mechanism involves increasing levels of itaconate and IRG1 (also known as NGFI-A) in the spinal cord after nerve injury. In addition, they found that administering 4-octyl itaconate, a derivative of itaconate, reduced nerve pain in both male and female rats. They also identified that 4-octyl itaconate treatment increased levels of IL-10 and activated the STAT3/β-endorphin pathway in the spinal cord, and the pain-relieving effect of itaconate was diminished in mice lacking IL-10.^15^

Considering that there is no complete information about the effects of DMI on the expression changes of genes NGFI-A and NGFI-B and inflammatory cytokines in the spinal cord following peripheral injection of formalin, the purpose of this study was to investigate the effect of DMI on expressions of NGFI-A and NGFI-B and inflammatory cytokines in the spinal cord in the formalin test.

## Materials and methods

### Animals

All experiments were carried out to minimize the suffering of animals. The rats could drink and eat freely (23 ± 1°C, 12 h of light and 12 h of darkness). This study was conducted following the protocols and guidelines approved by the Institutional Ethics Committee of Shahid Chamran University of Ahvaz, Ahvaz, Iran (code of ethics: EE/1401.2.24.226157/scu.ac.ir). It has been reported that this study has been conducted in accordance with the Animal Research: Reporting of In Vivo Experiments criteria.^16^

For this research, a total of 60 Wistar male rats weighing 250 ± 10 g were used, divided into five groups. The control group was given intraperitoneally injections of 0.9% NaCl, along with 50-μl subcutaneous injections of the same solution. The formalin group received intraperitoneally injections of 0.9% NaCl and subcutaneous injections of formalin 5% (solution in 0.9% NaCl).^17^ The DMI 10 mg/kg group received intraperitoneally injections of DMI 10 mg/kg^18^ (Sigma Chemical, St. Louis, Mo, USA) and subcutaneous injections of formalin 5%. The DMI 20 mg/kg group received intraperitoneally injections of DMI 20 mg/kg and subcutaneous injections of formalin solution 5%. Lastly, the diclofenac sodium group received intraperitoneally injections of diclofenac sodium 10 mg/kg and subcutaneous injections of formalin 5%. Peritoneal injections were administered 45 min prior to the formalin test.

### Pain model

The formalin test consists of two phases. Phase I occurs 5 min after formalin administration and results in acute peripheral pain. This pain is likely caused by the direct activation of nociceptors. The interphase follows, during which there is a period of analgesia. Finally, Phase II begins due to continuous inflammatory input and central pain sensitization. Each rat was placed in the formalin test chamber for 20 min to adapt to the new environment. After the administration of formalin in the hind paw, the animal's response (biting or licking the injected hind paw) to the nociceptive stimulus was recorded for 60 min.^19-21^

### Tissue sampling

The rats were anaesthetized with ketamine (40 mg/kg) and xylazine (5 mg/kg) and then sacrificed within 60 s.^22^ Two hours after formalin injection, spinal cord tissue from six rats was isolated to assessment of NGFI-A and NGFI-B genes. Additionally, samples were taken from six other rats 24 h later to investigate cytokines. The samples were kept at a temperature of −70°C until they were ready for further testing.

### Total RNA extraction, cDNA synthesis and real-time PCR

Firstly, the spinal cords were homogenized, and total RNA was extracted using the RNA extraction Kit (Parstous, Iran), following the manufacturer's instructions. The concentration of RNA was measured with the NanoDrop spectrophotometer. Then, a cDNA was synthesized from this RNA with the cDNA Synthesis Kit (Parstous, Iran). Primers were designed for rat GAPDH, NGFI-A, NGFI-B, TNF-α, IL-1β, IL6 and IL-10. To conduct the real-time PCR, the reactions were performed in a 20-µl volume, using the Sybr Green master mix kit (Parstous, Iran). The ratio of comparative expression of each gene was calculated between the treated and untreated samples as 2-(ΔΔCt). The sequence of the primers used can be found in Table 1.

**Table 1:** Primer sequences

Gene name	Sequence
GAPDH-rat-F	AGTTCAACGGCACAGTCAAG
GAPDH-rat-R	TACTCAGCACCAGCATCACC
NGFI-A-rat-F	AGCTGAGCTTTGGTTCTCCA
NGFI-A-rat-R	AGGTAACCGCAGCATTCCAA
NGFI-B-rat-F	TGCACTGTTCTCCGAGTTCT
NGFI-B-rat-R	AGGACACTTGCAACACCACT
IL-10-rat-F	AGTACAGCCGGGAAGACAATAA
IL-10-rat-R	CCTGCATTAAGGAGTCGGTTAG
TNF-α-rat-F	AGCCGATTTGCTATCTCATACCAG
TNF-α-rat-R	CCTTCACAGAGCAATGACTCCA
IL-1β-rat-F	CAACCAACAAGTTGATATTCTCCATG
IL-1β-rat-R	GATCCACACTCTCCAGCTGCA
IL-6-rat-F	ACGGCCTTCCCTACTTCACA
IL-6-rat-R	CATTTCCACGATTTCCCAGA

### Statistical analysis

We analysed the data using GraphPad Prism 8.0. Before proceeding, we conducted a normalization test based on the distribution and homogeneity of variances using the Kolmogorov–Smirnov test. Since the data were normal, we then used one-way ANOVA to evaluate the groups. We also conducted *post hoc* analyses using Tukey tests. A significance level of *P* < 0.05 was considered.

## Results

### Formalin test

**Figure 1:**
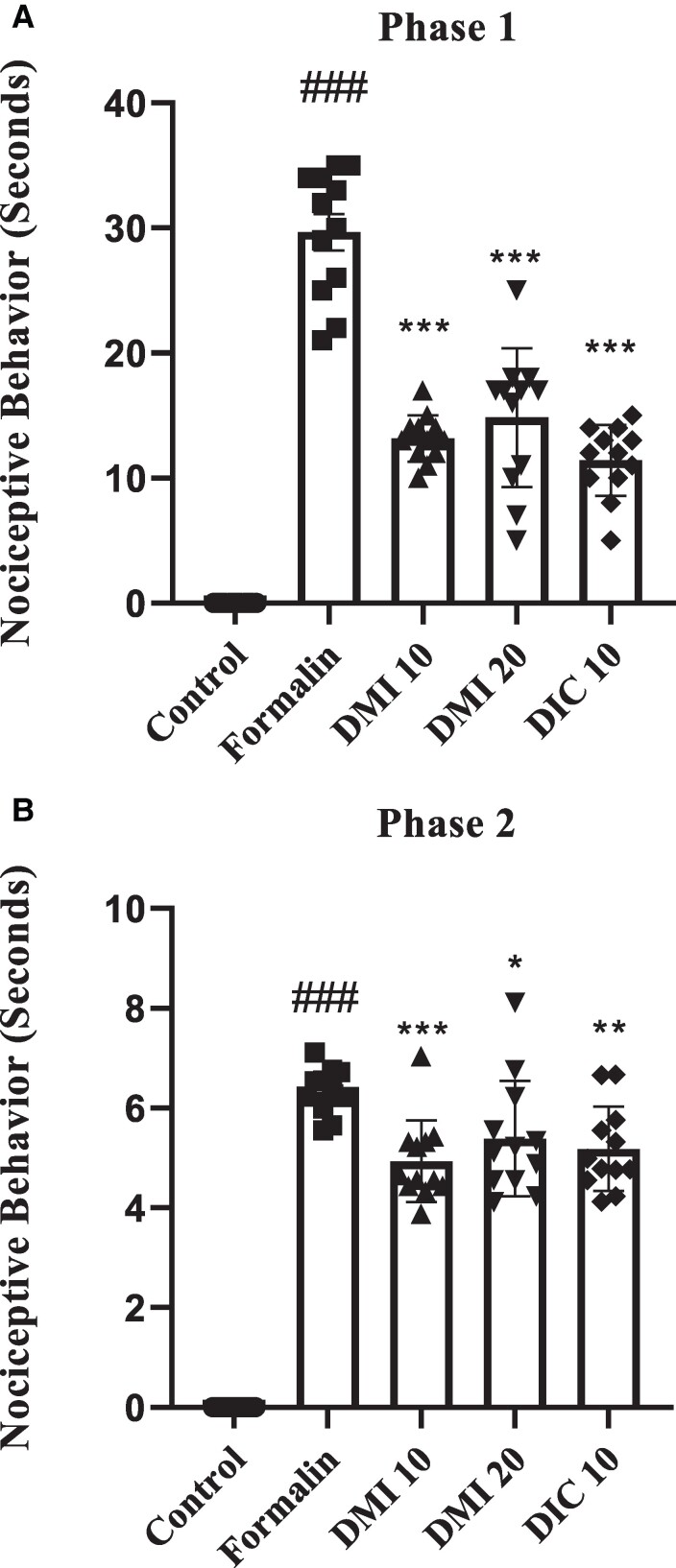
**The effect of DMI on hind paw licking time in the formalin test.** (**A)** Phase I of the formalin test. (**B**) Phase II of the formalin test. Data analysis using one-way ANOVA with *post hoc* Tukey tests. Data values are expressed as mean ± SEM. Each data point represents the data related to each animal. *n* = 12 rats/group. #*P* < 0.05, ##*P* < 0.01 and ###*P* < 0.001 formalin group versus the control group. *P < 0.05, **P < 0.01 and ***P < 0.001 DMI 10 mg/kg, DMI 20 mg/kg and diclofenac sodium 10 mg/kg groups versus the formalin group. DIC, diclofenac sodium; DMI, dimethyl itaconate.

In the first phase of the formalin test, the formalin group had significantly higher pain behavioural responses compared with the control group (*P* < 0.001). On the other hand, the groups that were given DMI 10, DMI 20 and diclofenac sodium 10 mg/kg had significantly lower pain behavioural responses than the formalin group (*P* < 0.001) (as shown in Fig. 1A).

In the second phase of the formalin test, the formalin group showed higher pain behavioural responses than the control group (*P* < 0.001). DMI 10 mg/kg, DMI 20 mg/kg and diclofenac 10 mg/kg groups lowered pain behavioural responses induced by formalin (*P* < 0.001, *P* < 0.05 and *P* < 0.01, respectively) (Fig. 1B).

### Expressions of the NGFI-A and NGFI-B mRNA

**Figure 2:**
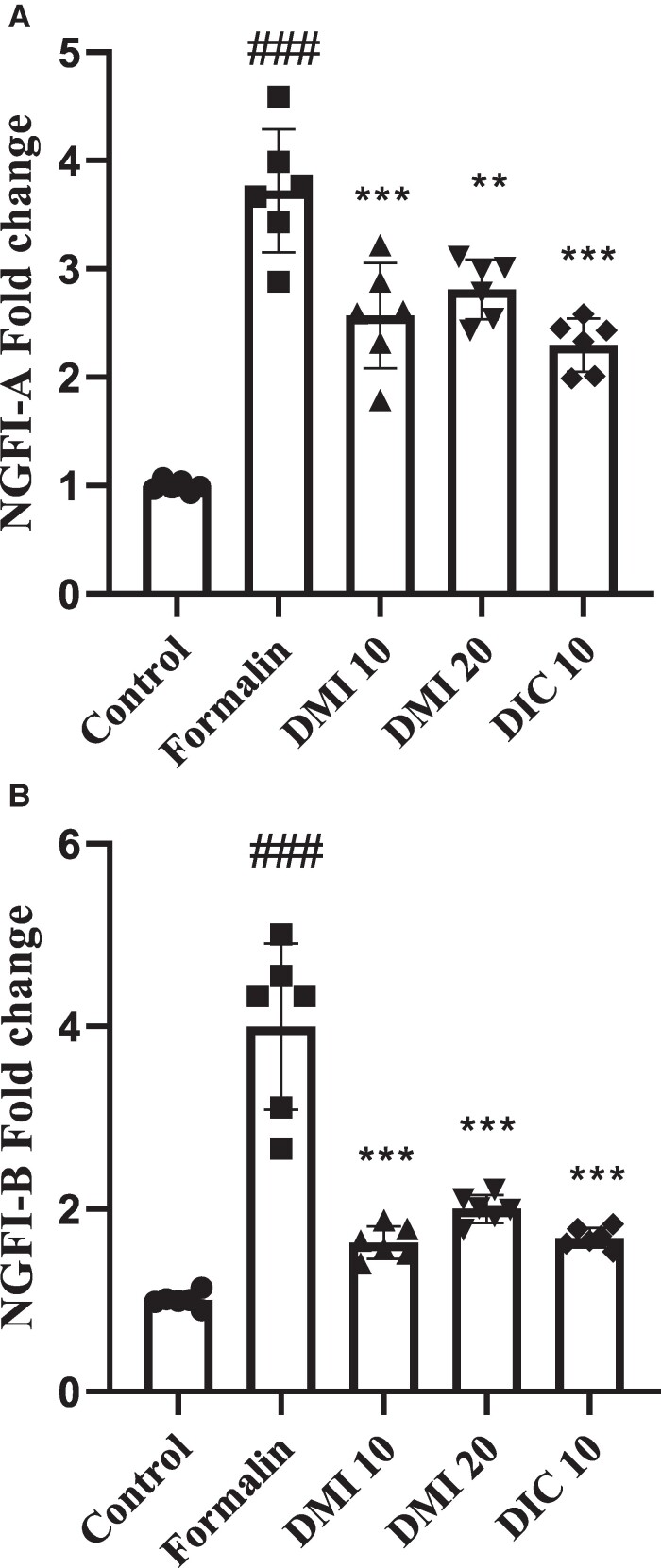
**The effect of DMI on the expression of (A) NGFI-A and (B) NGFI-B in spinal cord tissue in the formalin test.** Data analysis using one-way ANOVA with *post hoc* Tukey tests. Data values are expressed as mean ± SEM. *n* = 6 rats/group. #*P* < 0.05, ##*P* < 0.01 and ###*P* < 0.001 formalin group versus the control group. *P < 0.05, **P < 0.01 and ***P < 0.001 DMI 10 mg/kg, DMI 20 mg/kg and diclofenac sodium 10 mg/kg groups versus the formalin group. DIC, diclofenac sodium; DMI, dimethyl itaconate.

The NGFI-A gene expression in the group receiving formalin was higher than in the control group (*P* < 0.001). DMI 10 mg/kg, DMI 20 mg/kg and diclofenac sodium 10 mg/kg groups lowered NGFI-A expression induced by formalin (*P* < 0.001, *P* < 0.01 and *P* < 0.001, respectively) (Fig. 2A).

The NGFI-B gene was expressed more in the group treated with formalin compared with the control group (*P* < 0.001). Additionally, the expression of NGFI-B was lower in the DMI 10, DMI 20 and diclofenac sodium 10 groups than in the formalin group (*P* < 0.001) (Fig. 2B).

### Expressions of the IL-1β, TNF-α, IL-6 and IL-10

**Figure 3:**
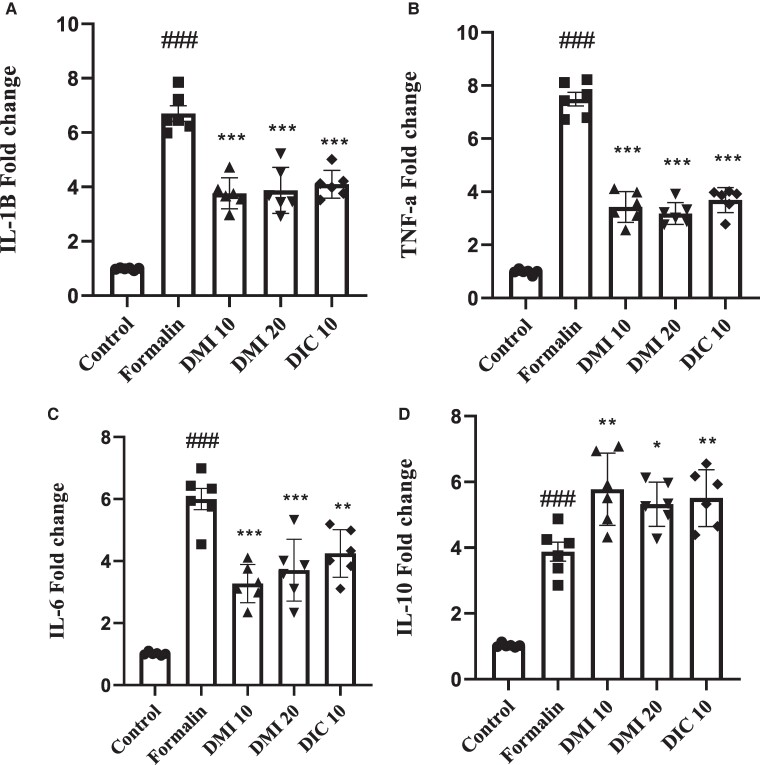
**The effect of DMI on the expression of cytokines in the spinal cord.** (**A**) IL-1β. (**B**) TNF-α. (**C**) IL-6. (**D**) IL-10. Data analysis using one-way ANOVA with *post hoc* Tukey tests. Data values are expressed as mean ± SEM. *n* = 6 rats/group. #*P* < 0.05, ##*P* < 0.01 and ###*P* < 0.001 formalin group versus the control group. *P < 0.05, **P < 0.01 and ***P < 0.001 DMI 10 mg/kg, and DMI 20 mg/kg and diclofenac sodium 10 mg/kg groups versus the formalin group. DIC, diclofenac sodium; DMI, dimethyl itaconate; IL-10, interleukin-10; IL-1β, interleukin-1 beta; IL-6, interleukin-6; TNF-α, tumor necrosis factor-alpha.

The IL-1β expression in the formalin group was higher than in the control group (*P* < 0.001). However, DMI 10, DMI 20 and diclofenac sodium 10 mg/kg groups had lower IL-1β expression in their spinal cords than the formalin group (*P* < 0.001) (Fig. 3A).

The TNF-α expression in the formalin group was higher than in the control group (*P* < 0.001). On the other hand, the DMI 10, DMI 20 and diclofenac sodium 10 mg/kg groups had lower TNF-α expression in their spinal cords than the formalin group (*P* < 0.001), as illustrated in Fig. 3B.

In this study, it was found that the IL-6 expression in the formalin group was higher than in the control group (*P* < 0.001). However, the DMI 10, DMI 20 and diclofenac sodium 10 mg/kg had lower IL-6 expression in their spinal cords than the formalin group. Again, the statistical analysis showed that the difference was significant (*P* < 0.001, *P* < 0.001 and *P* < 0.01). This information is presented in Fig. 3C.

The spinal cords of the formalin group had higher levels of IL-10 expression compared with the control group (*P* < 0.001). However, the groups treated with DMI 10, DMI 20 and diclofenac sodium 10 mg/kg had higher IL-10 expression in their spinal cords than the formalin group (*P* < 0.01, *P* < 0.05 and *P* < 0.01) (Fig. 3D).

## Discussion

In designing the current study, our primary interest was to investigate the expression of early response genes that altered in response to formalin injection in the spinal cord. The formalin injection increased the expressions of the NGFI-A and NGFI-B mRNA in the spinal cord (Fig. 2). In previous studies, it has been shown that formalin injection of the hind paw increased fast response genes such as NGFI-A and NGFI-B, jun B, c-jun, fos B and c-fos.^9,10,23^ Another importance related to measuring the expression of early response genes is the interpretation of the results. C-fos expression is often used to measure neuronal activation; however, inconsistent behavioural results with c-fos expression have been reported.^24^

NGF is a neurotrophic protein that has a multifunctional role in nociceptive processing. NGF modulates pain by altering ion channel activity, affecting the release of inflammatory mediators, regulating the expression of pain-related genes, and promoting local nerve sprouting.^25^ NGF induces of NGFI-A and NGFI-B.^26-28^ Activation of NGFI-A has reported after peripheral injury. The peripheral inflammation significantly was reduced in NGFI-A knockout mice. In addition, inflammation increases the expression of NGFI-A in the anterior cingulate cortex in the wild-type mice.^29^ NGFI-B antagonists improve pain intensity in mice models of neuropathic pain and bone cancer, so reducing NGFI-B could have analgesic effects.^30^ In the current study, the administration of 10 and 20 mg/kg DMI before formalin injection eliminated formalin-induced NGFI-A and NGFI-B expression in the spinal cord. DMI is produced by the mitochondrion-associated enzyme IRG1 and has recently been implicated as a regulator of macrophage activation. It has shown that DMI may cause analgesic effects through anti-inflammatory effects.^31-33^ DMI has also been reported to relieve persistent chronic pain by activating glial cells (such as microglia and astrocytes) in the spinal dorsal horn, increasing Nrf2 levels in the spinal cord and decreasing ERK1/2 phosphorylation.^34^

Our study found that, 24 h after formalin injection in the hind paw, the spinal cord showed an increase in the expression of cytokines IL-1β, TNF-α, IL-6 and IL-10 (Fig. 3). The activation of an inflammasome triggers the release of certain molecules in response, leading to a rise in pro-inflammatory cytokines and a decrease in anti-inflammatory cytokines due to local inflammatory stimulation. These cytokines can be transported retrogradely or via axonal or non-axonal mechanisms from the periphery to the dorsal root ganglion and dorsal horn, which can affect nerve activity and contribute to pain.^35^ Our research suggests that DMI can decrease the levels of IL-1β, TNF-α and IL-6 in the spinal cord after formalin administration. This is crucial because neuroinflammation is a significant factor in pain transmission at both spinal and supraspinal levels. Recent studies have revealed that the activation of glial cells and the release of inflammatory mediators play a vital role in central sensitization, resulting in pain.

IL-10 is antinociceptive in animal pain models without inducing tolerance. Its mechanism of action is believed to inhibit neuroinflammation.^36,37^ When IL-10 protein is administered systemically, it has been shown to produce pain relief in models of inflammatory pain, as measured by writhing in response to acetic acid or zymosan administered intraperitoneally,^38^ or by knee incapacitation after intra-articular injection of zymosan.^39^ Moreover, IL-10 protein has also been found to reduce pain-related behaviours in a model of spinal pain caused by the intraspinal injection of quisqualic acid.^39^ In our study, injecting formalin peripherally caused an increase in IL-10 levels in the spinal cord. However, DMI administration increased IL-10 levels in the spinal cord compared with the formalin group. Studies have shown that injecting formalin into the intraplantar region increases IL-10 levels in serum and specific brain regions, such as the dorsal root ganglion and spinal cord.^40,41^

The immune system and pain have a reciprocal relationship.^42^ Studies have shown that pro-inflammatory cytokines can cause or worsen inflammation and pain. Blocking their production or release may be an effective treatment strategy for pathological pain.^43^

We reported that IP injection of DMI exhibits an antinociceptive effect on formalin test in rats. We showed that DMI decreased the expression of NGFI-A and NGFI-B in the spinal cord. In addition, DMI increased IL-10 and decreased IL-1β, TNF-α and IL-6 in the spinal cord compared with the formalin group.

## Data Availability

The data are available upon access reasonable request.
